# Identification of High-Risk Patients With Nonalcoholic Fatty Liver Disease Using Noninvasive Tests From Primary Care and Endocrinology Real-World Practices

**DOI:** 10.14309/ctg.0000000000000340

**Published:** 2021-04-06

**Authors:** Zobair M. Younossi, Huong Pham, Sean Felix, Maria Stepanova, Thomas Jeffers, Elena Younossi, Hussain Allawi, Brian Lam, Rebecca Cable, Mariam Afendy, Zahra Younoszai, Arian Afendy, Nila Rafiq, Nahrain Alzubaidi, Yasser Ousman, Marc Bailey, Zik Chris, Maria Castillo-Catoni, Pratima Fozdar, Maria Ramirez, Mehreen Husain, Evis Hudson, Ingrid Schneider, Pegah Golabi, Fatema Nader

**Affiliations:** 1 Betty and Guy Beatty Center for Integrated Research, Inova Health System, Falls Church, Virginia, USA;; 2 Inova Medicine, Inova Health System, Falls Church Virginia, USA;; 3 Center for Outcomes Research in Liver Disease, Washington, DC, USA;; 4 Department of Medicine, Center for Liver Disease, Inova Fairfax Medical Campus, Falls Church, Virginia, USA.

## Abstract

We aimed to identify high-risk nonalcoholic fatty liver disease (NAFLD) patients seen at the primary care and endocrinology practices and link them to gastrohepatology care.

**METHODS:**

Using the electronic health record, patients who either had the diagnosis of type 2 diabetes or had 2 of 3 other metabolic risk factors met criteria for inclusion in the study. Using noninvasive fibrosis tests (NITs) to identify high risk of fibrosis, patients who met the NIT prespecified criteria were referred to gastrohepatology for clinical assessment and transient elastography.

**RESULTS:**

From 7,555 patients initially screened, 1707 (22.6%) met the inclusion criteria, 716 (42%) agreed to enroll, and 184 (25.7%) met the prespecified NIT criteria and eligibility for linkage to GE-HEP where 103 patients (68 ± 9 years of age, 50% men, 56% white) agreed to undergo linkage assessments. Their NIT scores were APRI of 0.38 ± 0.24, FIB-4 of 1.98 ± 0.87, and NAFLD Fibrosis Score of 0.36 ± 1.03; 68 (66%) linked patients had controlled attenuation parameter >248 dB/m, 62 (60%) had liver stiffness <6 kPa, and 8 (8%) had liver stiffness >12 kPa. Liver stiffness for the overall group was 6.7 ± 4.2 kPa, controlled attenuation parameter 282 ± 64 dB/m, and FAST score 0.22 ± 0.22. Linked patients with presumed advanced fibrosis had significantly higher body mass index (36.4 ± 6.6 vs 31.2 ± 6.4 kg/m^2^, *P* = 0.025) and higher NIT scores (APRI 0.89 ± 0.52 vs 0.33 ± 0.14, FIB-4 3.21 ± 2.06 vs 1.88 ± 0.60, and NAFLD Fibrosis Score 1.58 ± 1.33 vs 0.25 ± 0.94).

**DISCUSSION:**

By applying a simple prespecified multistep algorithm using electronic health record with clinical risk factors and NITs followed by transient elastography, patients with nonalcoholic fatty liver disease seen in PCP and ENDO practices can be easily identified.

## INTRODUCTION

Nonalcoholic fatty liver disease (NAFLD) is the most common cause of chronic liver disease, affecting at least 25% of the general population in most regions of the world (1,2). NAFLD is defined as at least 5% fat deposition in the hepatic parenchyma in the absence of other causes of fatty liver or chronic liver disease (3,4). NAFLD is a heterogeneous disease and includes nonalcoholic steatohepatitis (NASH) which requires not only hepatic steatosis but also evidence of hepatocyte injury such as hepatocyte ballooning (3,4). The spectrum of NAFLD is associated with components of metabolic syndrome, including obesity, type 2 diabetes (T2DM), hypertension, and dyslipidemia (5). Although the exact rates are not known, it has been suggested that 12%–20% of patients with NAFLD have underlying NASH with an estimated prevalence of 1.5%–6.5% in the general population (2,6).

The heterogeneity of NAFLD is not only reflected in the spectrum of histopathologic changes or the diversity of underlying its pathogenic pathways but also in terms of the progressiveness of the subtypes of NAFLD. In this context, evidence suggests that NASH is the subtype that can potentially progress to cirrhosis and its complications. In fact, it is estimated that 15%–20% of patients with NASH can progress to cirrhosis (1–7). Although a relatively small proportion of patients with NAFLD are at risk of progressive liver disease, all patients with NAFLD are at increased risk of cardiovascular disease and associated mortality (8). Indeed, longitudinal studies of NAFLD have demonstrated an increase in both cardiovascular and overall mortality (9,10). By contrast, increased liver-related mortality seems to be limited to those with underlying NASH (9–15).

The enormous burden of NAFLD and NASH was recently estimated by modeling study that suggested around 16.5 million cases of NASH in the United States (7). In this context, the published recent evidence supports this increasing burden. In fact, NAFLD is now the main driver of cirrhosis, HCC, or being listed for liver transplantation in the United States (16,17). In addition, increasing data suggest that the future global burden of liver disease is highly likely to be driven by NAFLD and its complications (cirrhosis and HCC) (18,19).

As the understanding of the global burden of NAFLD grows, factors that may predict adverse outcomes in NASH have been examined. In this context, presence of T2DM is the most important clinical driver of mortality in patients with NAFLD (11). NAFLD patients with T2DM seem to have higher risk of mortality which is independent of other clinical variables (11–13). In addition to the clinical predictors of mortality, histologic stage of fibrosis indicated by a liver biopsy has been shown to independently predicts liver-related and all-cause mortality in NAFLD (9,10,14,15).

Despite the evidence supporting the growing burden of NAFLD, identification of these patients and linking those who are at high risk of adverse outcomes to preventative care and treatment are lacking (20). This is especially important for patients with metabolic syndrome who are commonly managed by primary care and endocrinology practices. In this context, screening patients from primary care and endocrinology practices and determining which patients with NAFLD are the highest risk of adverse outcome will be essential. As noted previously, the clinical risk profile of patients such as presence of T2DM or other components of metabolic syndrome and histologic stage of fibrosis can provide some clues about patients with NAFLD who are at risk of adverse outcomes (11–15,21). Despite the importance of histologic stage of hepatic fibrosis, it is impractical to expect large volumes of liver biopsies in the clinical practice. Given that, replacing histologic staging with noninvasive tests (NITs) for fibrosis will be of great interest in the clinical practice (22–24).

Noninvasive tests for assessment of fibrosis in NAFLD can be broadly categorized into serum biomarker-based scores and imaging-based assessment of liver stiffness (24). Some of these NITs can be easily calculated using routine clinical and laboratory data (22,23). An important point-of-care imaging test to assess liver stiffness is transient elastography (TE) (24). This technique is being used by a large number of gastroenterology and hepatology practices to identify patients with NAFLD who are at risk of significant liver stiffness as a surrogate of hepatic fibrosis (22–24). Although each single NIT has limited accuracy to risk stratify patient with NAFLD, it is plausible that clinical profiles, serum NITs, and TE can be used in combination to develop better algorithms that can be implemented in real-world clinical practices to identify high-risk NAFLD patients. Therefore, the aim of our study was to identify patients with NAFLD who are at the highest risk of adverse outcomes and to link them to appropriate care with the aim to optimize their clinical care and their ability to have access to cutting edge monitoring and treatment protocols.

## METHODS

### Study population

We contacted affiliated primary care offices (PCP) and endocrinology outpatient offices (ENDO) to screen patients for risk of NAFLD. A 2-step screening method was performed to identify high-risk NAFLD patients and link them to gastroenterology and hepatology (GE-HEP) clinics.

For the initial screening, study inclusion criteria required having 1 of the following: 1) presence of established T2DM and 1 other component of metabolic syndrome (hypertension, hyperlipidemia, or body mass index [BMI] > 29.9) or (ii) T2DM with elevated aspartate aminotransferase (AST) or alanine aminotransaminase (ALT) levels (1.5 × upper limit of normal) or history of fatty liver by any imaging modality; and 3) in the absence of established T2DM, presence of 3 components of metabolic syndrome (hypertension, hyperlipidemia, and BMI > 29.9). Main exclusion criteria were other causes of chronic liver disease and inability/unwillingness to provide consent.

Study staff, in conjunction with participating Endocrinology and Primary Care practices, screened individuals for the inclusion/exclusion criteria using their electronic medical record. All patients who were considered eligible for participation based on the screening were confirmed by their respective primary providers. Patients were then contacted and invited to participate by telephone. For those who agreed to participate, inclusion/exclusion criteria were reviewed and institutional review board-approved phone consent was administered. After informed consent, clinical and demographic data were collected using a prespecified data collection form; collected parameters included age, sex, ethnicity, BMI, comorbidities, and laboratory results. Based on these data, NITs were used to determine high-risk patients.

### Detection of high-risk patients and linkage to care

Using laboratory and clinical data, 3 NITs (AST-to-platelet ratio [APRI], NAFLD Fibrosis Score [NFS], and FIB-4 index) were calculated (23,25–27). For NFS, 2 cutoff points were selected to identify presence (>0.67) and absence (<−1.45) of significant fibrosis. In terms of the FIB-4 index, we assumed that a value < 1.45, presence of advanced hepatic fibrosis could be excluded with 90%–98% certainty, and for a threshold value of >3.25 for the FIB-4 index, leads to a positive predictive value of 53%–75% for advanced fibrosis. On the other hand, sensitivity and specificity of an APRI score >1 for significant fibrosis is 30% and 92.8%, respectively. The criterion for high-risk NAFLD (presumed high-risk NASH) was to have at least 2 NITs above certain thresholds (APRI >1.0, NFS > −1.45, and FIB-4 > 1.45). Participants who fulfilled this criterion were regarded as high-risk NASH and were eligible for the linkage-to-care step. If eligible subjects agreed to participate in the linkage step, they were referred to a gastroenterology or hepatology clinic. Patients were seen in person, and medical history was collected followed by physical examination, blood sample collection, and TE (FibroScan mini 430; Echosens, Waltham, MA).

As detailed in the manufacturer's guidance and training, the TE procedure was begun using the medium probe (M probe). If the FibroScan machine indicated that the M probe was not able to obtain adequate measurements due to increased body habitus, the extra-large probe (XL probe) was used. At least 10 adequate measurements were obtained in standard fashion per established manufacturer guidelines, and the median stiffness and controlled attenuation parameter scores were automatically calculated by the FibroScan. Patients with liver stiffness of less than 6 kPa were presumed to have no significant hepatic fibrosis, patients with liver stiffness of ≥8 kPa or more were presumed to have clinically significant fibrosis, and patients with ≥12 kPa were considered to have advanced fibrosis or cirrhosis.

### Statistical analysis

All collected demographic and clinical parameters were summarized as mean with SD or median with interquartile range (IQR). Chi-square and Mann-Whitney statistical tests were used for comparison of the parameters between subgroups of interest. All statistical analyses were run using SAS 9.4 (SAS Institute, Cary, NC).

## RESULTS

A total of 7,555 patients were screened for the study. Among those, the mean age was 54 years, 38% male, 51% white, and 26% were black. In terms of comorbidities, 26% had T2DM, 48% had hypertension, and 56% had hyperlipidemia; mean BMI was 29.9. Of these patients, 1,389 (18.4%) had insufficient data to determine inclusion criteria and 4,457 (59%) did not meet the initial criteria for the study. Therefore, a total of 1,707 (22.6%) participants met the initial inclusion criteria. Among them, 716 (42%) agreed to proceed with the 2nd step of screening by NITs to determine presence of high-risk scores (Table 1). Based on this, 184 patients were eligible for linkage to care, while 103 participants agreed to be linked to GE-HEP for clinical assessment and TE.

**Table 1.** Clinicodemographic characteristics of patients linked to care

Table	Linked	Not linked	Prob	All called
N	103	613		716
Eligible	103 (100.0%)	81 (13.2%)	<0.0001	184 (25.7%)
Age	68.3 ± 9.6	58.3 ± 13.1	<0.0001	59.7 ± 13.1
Male sex	53 (51.5%)	247 (40.3%)	0.0336	300 (41.9%)
Race				
White	60 (58.3%)	325 (53.3%)	0.35	385 (54.0%)
Black	30 (29.1%)	181 (29.7%)	0.91	211 (29.6%)
Hispanic	4 (3.9%)	45 (7.4%)	0.19	49 (6.9%)
Asian	5 (4.9%)	42 (6.9%)	0.44	47 (6.6%)
BMI, kg/m^2^	31.4 ± 6.6	33.2 ± 7.7	0.041	33.0 ± 7.5
Diabetes	56 (54.4%)	266 (43.4%)	0.0383	322 (45.0%)
Hyperlipidemia	91 (88.3%)	527 (86.0%)	0.52	618 (86.3%)
Hypertension	85 (82.5%)	507 (82.7%)	0.96	592 (82.7%)
History of myocardial infarction	9 (8.9%)	25 (4.1%)	0.0354	34 (4.8%)
History of stroke	4 (4.0%)	27 (4.4%)	0.83	31 (4.4%)
History of congestive heart failure	1 (1.0%)	14 (2.3%)	0.39	15 (2.1%)
History of cancer	24 (23.5%)	89 (14.5%)	0.0213	113 (15.8%)
APRI	0.419 ± 0.282	0.219 ± 0.108	<0.0001	0.247 ± 0.162
Fibrosis-4 (FIB-4)	2.07 ± 0.80	1.06 ± 0.51	<0.0001	1.20 ± 0.67
NAFLD Fibrosis Score	0.382 ± 0.996	−1.11 ± 1.42	<0.0001	−0.893 ± 1.464

APRI, AST-to-platelet ratio; AST, aspartate aminotransferase; BMI, body mass index; NAFLD, nonalcoholic fatty liver disease.

### Clinicodemographic characteristics of patients linked to care

Among 103 patients who agreed with linkage-to-care and underwent TE (Table 1), mean age was 68.3 ± 9.6 years, 51.5% male, 58.3% white, 29.1% black, 3.9% Hispanic, and 4.9% Asian. In terms of comorbidities, 54.4% had T2DM, 82.5% had hypertension, 88.3% had hyperlipidemia, 8.9% had a history of myocardial infarction (MI), 4% had stroke, 23.5% had a history of cancer, and mean BMI was 31.4 ± 6.6. Among noninvasive tests, mean APRI was 0.419 ± 0.282, mean FIB-4 index 2.07 ± 0.80, and mean NFS 0.382 ± 0.996.

As FIB-4 index and NFS include age as variable, we performed a propensity match analysis by adjusting for age and sex (Supplementary Table 1, http://links.lww.com/CTG/A566). Even after this adjustment, compared with patients who were not linked, linked patients had significantly higher APRI (0.419 vs 0.214), FIB-4 index (2.07 vs 1.23), and NFS (0.382 vs −0.514, all *P* < 0.001).

### Comparison of patients based on fibrosis severity by transient elastography

Among 103 patients who underwent TE, 60.2% had liver stiffness <6 kPa, 22.3% between 6 and 8 kPa, 5.8% between 8 and 10 kPa, 3.9% between 10 and 12 kPa, and 7.8% had >12 kPa (Table 2).

**Table 2.** Distribution of patients based on fibrosis severity by transient elastography

	All
N	103
Liver stiffness < 6 kPa	62 (60.2%)
Liver stiffness 6–8 kPa	23 (22.3%)
Liver stiffness 8–10 kPa	6 (5.8%)
Liver stiffness 10–12 kPa	4 (3.9%)
Liver stiffness ≥12 kPa	8 (7.8%)

kPa, kilopascal.

Compared with patients with liver stiffness of <8 kPa, patients with liver stiffness of ≥8 kPa on TE were younger (mean age 61.3 vs 69.9 years) and had higher mean BMI (36.5 vs 30.5), higher controlled attenuation parameter (318 vs 274 dB/m^2^), AST (44 vs 25 U/L), ALT (51 vs 23 U/L), and alkaline phosphatase (95 vs 76 U/L) levels, and higher APRI (0.665 vs 0.316) (all *P* < 0.05) but similar FIB-4 index (2.36 vs 1.90) and NFS (0.705 vs 0.281) (*P* > 0.05) (Table 3).

**Table 3.** Clinicodemographic characteristics of patients based on fibrosis severity by transient elastography

Table	Liver stiffness > 8 kPa) (N = 18)	Liver stiffness ≤ 8 kPa) (N = 85)	*P*	All linked patients (N = 103)
Age	61.3 ± 9.2	69.9 ± 8.8	**0.0011**	68.4 ± 9.4
Male sex	8 (44.4%)	43 (50.6%)	0.64	51 (49.5%)
Race white	12 (66.7%)	46 (54.1%)	0.33	58 (56.3%)
Black	3 (16.7%)	26 (30.6%)	0.23	29 (28.2%)
Hispanic	2 (11.1%)	2 (2.4%)	0.08	4 (3.9%)
Asian	1 (5.6%)	4 (4.7%)	0.88	5 (4.9%)
Other	0 (0.0%)	7 (8.2%)	0.21	7 (6.8%)
Body mass index, kg/m^2^	36.5 ± 7.1	30.5 ± 6	**0.0014**	31.6 ± 6.5
History of hyperlipidemia	15 (83.3%)	80 (94.1%)	0.12	95 (92.2%)
History of hypertension	12 (66.7%)	76 (89.4%)	**0.0129**	88 (85.4%)
Diabetes	13 (72.2%)	44 (51.8%)	0.11	57 (55.3%)
Depression	2 (11.1%)	15 (17.6%)	0.50	17 (16.5%)
History of sleep apnea	5 (27.8%)	19 (22.4%)	0.62	24 (23.3%)
History of cardiovascular disease	4 (22.2%)	12 (14.3%)	0.40	16 (15.7%)
History of myocardial infarction	2 (11.1%)	4 (4.9%)	0.31	6 (6.0%)
History of stroke	0 (0.0%)	4 (4.8%)	0.34	4 (3.9%)
Current smoker	2 (11.1%)	4 (4.7%)	0.29	6 (5.8%)
Exercise > 90 min/wk	7 (38.9%)	38 (46.9%)	0.54	45 (45.5%)
Albumin, g/dL	4.13 ± 0.35	4.07 ± 0.34	0.47	4.08 ± 0.34
Alanine aminotransferase, IU/L	50.7 ± 34.0	23.4 ± 15.4	**<0.0001**	28.2 ± 22.3
Aspartate aminotransferase, IU/L	44.4 ± 22.7	24.7 ± 9.9	**<0.0001**	28.1 ± 14.9
Total bilirubin, mg/dL	0.71 ± 0.29	0.64 ± 0.27	0.40	0.65 ± 0.27
Glucose mg/dL	135.8 ± 58.6	117.6 ± 43.4	0.13	120.8 ± 46.6
Hemoglobin A1c (HbA1c), %	8.17 ± 1.72	7.14 ± 1.59	**0.0258**	7.32 ± 1.65
Hemoglobin, gr/dL	14.1 ± 1.3	13.8 ± 1.5	0.46	13.9 ± 1.5
Platelets, 10^3/UL	182.8 ± 46.3	202.6 ± 45.6	0.17	199.1 ± 46.1
Total cholesterol, mg/dL	166.7 ± 41.5	169.2 ± 36.4	0.81	168.9 ± 36.9
Low-density lipoprotein mg/dL	95.9 ± 43.8	93.8 ± 30.7	0.79	94.1 ± 32.7
High-density lipoprotein, mg/dL	44.7 ± 11.0	52.1 ± 13.9	0.08	51.0 ± 13.7
Triglycerides, mg/dL	142.3 ± 56.3	121.6 ± 89.4	0.07	124.7 ± 85.3

BMI, body mass index.

Compared with noncirrhotic patients (liver stiffness <12 kPa), patients with presumed cirrhosis (liver stiffness ≥12 kPa had higher mean BMI (36.4 vs 31.2 years), AST (53 vs 26 U/L), ALT (59 vs 26 U/L), and alkaline phosphatase (116 vs 76 U/L) levels, higher APRI (0.894 vs 0.333), FIB-4 index (3.21 vs 1.88), NFS (1.580 vs 0.252), and were more likely to have diabetes (100% vs 51.6%), history of CVD (50% vs 12.8%), and MI (25% vs 4.3%) (all *P* < 0.05).

## DISCUSSION

In the recent decades, NAFLD and its progressive form NASH have become the fastest growing causes of chronic liver disease contributing to its global burden (16,28,29). This tremendous burden of NAFLD is related to its very high prevalence in the general population and some potential for progression in those with NASH. In this context, recent models have suggested that there are 73 million patients with NAFLD and 9 million patients with NASH in the United States (7). Furthermore, the same models have predicted that between 2015 and 2030, the number of cases of advanced NASH in the United States will double, leading to increased liver-related complications and approximately 800,000 extra deaths (7).

In this context, it is important to note that most patients with NAFLD have likely not been identified and most of them are presumably being seen in the primary care practices without being diagnosed. It is important to note that not all patients with NAFLD will progress to cirrhosis or its complications (30). In fact, only 10%–15% of those with NASH may progress to cirrhosis. Nevertheless, identifying patients with NAFLD who are at risk of adverse outcomes will be critical to address the increasing burden of NAFLD. On the other hand, the sheer number of patients with NAFLD and NASH can overwhelm any clinical practice. Therefore, efforts to identify high-risk patients with NAFLD must be aided by development of efficient and easily applicable algorithms that can be used by clinicians in their daily practice. In this context, our study prospectively implemented a simple algorithm using easily available NITs (FIB-4, NFS, and APRI) for linking these patients with a potentially higher risk of progression to further specialty care, including clinical assessment and TE.

Our study demonstrated that 60% of patients with high-risk NIT scores had liver stiffness <6 kPa by TE and can be considered at minimal risk of adverse outcomes. These patients can be redirected back to their primary care providers to optimize their cardiometabolic risks to avert future cardiovascular complications. By contrast, 18% of the participants with high NIT scores also had elevated liver stiffness (TE ≥ 8 kPa), which indicates some degree of hepatic fibrosis and potential for progressive liver disease. These patients also had higher BMI and elevated liver enzyme levels which are consistent with the current literature (27–29). Interestingly, these patients were significantly younger which may be worrisome as they have longer life span to potentially progress to cirrhosis.

Finally, our data showed that 8% of the screened cohort had liver stiffness ≥12 kPa, indicating possible cirrhosis. Again, compared with noncirrhotic individuals, patients with cirrhosis had higher BMI, liver enzyme levels, and NIT scores and were more likely to have comorbidities including diabetes and cardiovascular disease. These are also consistent with the profile of patients with NAFLD at risk of progressive liver disease (29,31). Early identification of these patients can lead not only to possibly stricter lifestyle modifications with the aim to reduce hepatic damage but also to the need for assessment for complications of cirrhosis such as HCC and esophageal varices.

The main strength of the current study is the prospective application of a stepwise algorithm in real-world practices for detection of patients who had the highest risk of NAFLD. Although liver biopsy remains the gold standard for detecting and grading liver fibrosis, it has multiple caveats and cannot be used in a large scale in the clinical practice (32). In this context, the lack of liver biopsy can also be considered as a potential limitation of this study because some patients who are identified as no significant fibrosis may actually have hepatic fibrosis if a liver biopsy were to be performed. We believe the multistep approach of using our algorithm minimizes this limitation. Finally, our study was limited to 10 primary care and gastroenterology clinics in a relatively small area, thus making it difficult to generalize to the population level.

In summary, this study demonstrates that a stepwise prospective application of an algorithm using NITs and TE in clinical practice setting can lead to identification of patients with high-risk NAFLD. Further studies are needed to use this or other similar algorithms in clinical practice using electronic health records.

## CONFLICTS OF INTEREST

**Guarantor of the article:** Zobair M. Younossi, MD, MPH.

**Specific author contributions:** Z.Y., M.S., N.A., Y.O., M.B., Z.C., M.C-C., P.F., M.R., M.H., E.H., and F.N.: study design. H.P., S.F., M.S., T.J., E.Y., H.A., B.L., R.C., M.A., Z.Y., A.A., N.R., and I.S.: data collection. M.S.: data analysis. M.S., P.G., B.L., and N.R.: interpretation of data. P.G., M.S., B.L., and T.J.: drafting of the manuscript. Z.M.Y.: critical revision of the manuscript for important intellectual content. Z.M.Y.: study supervision. All authors read and approved the final version of the manuscript.

**Financial support:** This research did not receive any specific grant from funding agencies in the public, commercial, or not-for-profit sectors.

**Potential competing interests:** ZMY has received research funds or served as consultant to Gilead Sciences, Intercept, NovoNordisk, BMS, AbbVie, Merck, Madrigal, Genfit, Siemens, BMS, Terns, and Viking. All other authors have no conflict of interest to disclose.

**Study Highlights**

**WHAT IS KNOWN**

✓ Identification of patients with nonalcoholic fatty liver disease (NAFLD) at high risk of adverse outcomes with linkage to specialty care remain suboptimal.
✓ Patients with metabolic syndrome are at high risk of developing NAFLD.
✓ Most patients with metabolic syndrome are seen in primary care or endocrinology practices.

**WHAT IS NEW HERE**

✓ A 2-step algorithm for referral was developed to assist frontline care providers in identifying patients with NAFLD and patients with NAFLD at high risk of adverse outcomes.
✓ Initial step was identification of NAFLD based on presence of type 2 diabetes with 1 other metabolic component of MetS, or having 3 metabolic components of MetS (obesity, hypertension, and hyperlipidemia) or having type 2 diabetes with elevated aspartate aminotransferase or alanine aminotransaminase levels (1.5 × upper limit of normal) or history of fatty liver by any imaging modality
✓ Ten percent of referred patients had kPa > 8 < 12 by transient elastography and were found to be younger with higher body mass index and elevated liver enzymes.
✓ Eight percent had liver stiffness ≥12 kPa, indicating possible cirrhosis, and had higher body mass index, liver enzyme levels, and noninvasive fibrosis test scores, and were more likely to have comorbidities including diabetes and cardiovascular disease.

**TRANSLATIONAL IMPACT**

✓ Almost 20% of referred patients had evidence of increased liver stiffness with almost 10% possibly having cirrhosis. Because of the number of patients who may have NAFLD, this percent of patients with liver stiffness could still indicate a large number of patients with adverse outcomes related to NAFLD.
✓ Attention needs to continue to focus on addressing metabolic risk factors which are now evident among the younger population to decrease the risk of the development of cardiovascular disease and NAFLD-related fibrosis.

## Supplementary Material

SUPPLEMENTARY MATERIAL

## References

[1] Younossi ZM Koenig AB Abdelatif D. Global epidemiology of nonalcoholic fatty liver disease-Meta-analytic assessment of prevalence, incidence, and outcomes [Internet]. Hepatology 2016;64:73–84. 26707365 10.1002/hep.28431

[2] Younossi Z Anstee QM Marietti M. Global burden of NAFLD and NASH: Trends, predictions, risk factors and prevention [internet]. Nat Rev Gastroenterol Hepatol 2018;15:11–20. 28930295 10.1038/nrgastro.2017.109

[3] Bondini S Kleiner DE Goodman ZD. Pathologic assessment of non-alcoholic fatty liver disease [Internet]. Clin Liver Dis 2007;11:17–23, vii. 17544969 10.1016/j.cld.2007.02.002

[4] Younossi ZM Stepanova M Rafiq N. Pathologic criteria for nonalcoholic steatohepatitis: Interprotocol agreement and ability to predict liver-related mortality [internet]. Hepatology 2011;53:1874–82. 21360720 10.1002/hep.24268

[5] Golabi P Otgonsuren M Avila Lde. Components of metabolic syndrome increase the risk of mortality in nonalcoholic fatty liver disease (NAFLD) [Internet]. Medicine 2018;97:e0214. 29595666 10.1097/MD.0000000000010214 PMC5895395

[6] Younossi ZM. Non-alcoholic fatty liver disease-A global public health perspective [internet]. J Hepatol (10.1016/j.jhep.2018.10.033. 30414863

[7] Estes C Razavi H Loomba R. Modeling the epidemic of nonalcoholic fatty liver disease demonstrates an exponential increase in burden of disease [Internet]. Hepatology 2018;67:123–33. 28802062 10.1002/hep.29466 PMC5767767

[8] Byrne CD Targher G. Nafld: A multisystem disease [internet]. J Hepatol 2015;62:S47–64. 25920090 10.1016/j.jhep.2014.12.012

[9] Dulai PS Singh S Patel J. Increased risk of mortality by fibrosis stage in nonalcoholic fatty liver disease: Systematic review and meta-analysis [Internet]. Hepatology 2017;65:1557–65. 28130788 10.1002/hep.29085 PMC5397356

[10] Stepanova M Rafiq N Makhlouf H. Predictors of all-cause mortality and liver-related mortality in patients with non-alcoholic fatty liver disease (NAFLD) [Internet]. Dig Dis Sci 2013;58:3017–23. 23775317 10.1007/s10620-013-2743-5

[11] Younossi ZM Gramlich T Matteoni CA. Nonalcoholic fatty liver disease in patients with type 2 diabetes [Internet]. Clin Gastroenterol Hepatol 2004;2:262–5. 15017611 10.1016/s1542-3565(04)00014-x

[12] Younossi ZM Golabi P Avila Lde. The global epidemiology of NAFLD and NASH in patients with type 2 diabetes: A systematic review and meta-analysis [internet]. J Hepatol (10.1016/j.jhep.2019.06.021) (2019). Accessed September 21, 2020. 31279902

[13] Younossi ZM Tampi RP Racila A. Economic and clinical burden of nonalcoholic steatohepatitis in patients with type 2 diabetes in the United States [internet]. Diabetes Care (10.2337/dc19-1113) (2019). Accessed September 21, 2020. 31658974

[14] Ekstedt M Hagström H Nasr P. Fibrosis stage is the strongest predictor for disease-specific mortality in NAFLD after up to 33 years of follow-up [Internet]. Hepatology 2015;61:1547–54. 25125077 10.1002/hep.27368

[15] Angulo P Kleiner DE Dam-Larsen S. Liver fibrosis, but No other histologic features, is associated with long-term outcomes of patients with nonalcoholic fatty liver disease [internet]. Gastroenterology 2015;149:389–97.e10. 25935633 10.1053/j.gastro.2015.04.043 PMC4516664

[16] Wong RJ Aguilar M Cheung R. Nonalcoholic steatohepatitis is the second leading etiology of liver disease among adults awaiting liver transplantation in the United States [Internet]. Gastroenterology 2015;148:547–55. 25461851 10.1053/j.gastro.2014.11.039

[17] Younossi Z Stepanova M Ong JP. Nonalcoholic steatohepatitis is the fastest growing cause of hepatocellular carcinoma in liver transplant candidates [internet]. Clin Gastroenterol Hepatol (10.1016/j.cgh.2018.05.057) (2018). Accessed September 21, 2020. 29908364

[18] Paik JM Golabi P Younossi Y. Changes in the global burden of chronic liver diseases from 2012 to 2017: The growing impact of nonalcoholic fatty liver disease [internet]. Hepatology (10.1002/hep.31173) (2020). Accessed September 21, 2020. 32043613

[19] Younossi ZM Henry L Bush H. Clinical and economic burden of nonalcoholic fatty liver disease and nonalcoholic steatohepatitis [internet]. Clin Liver Dis 2018;22:1–10. 29128049 10.1016/j.cld.2017.08.001

[20] Augustin S Ahmed A Alkhouri N. Identification of patients with advanced fibrosis due to nonalcoholic fatty liver disease: Considerations for best practice [internet]. J Gastrointestin Liver Dis 2020;29:235–45. 32530991 10.15403/jgld-775

[21] Younossi ZM Otgonsuren M Venkatesan C. In patients with non-alcoholic fatty liver disease, metabolically abnormal individuals are at a higher risk for mortality while metabolically normal individuals are not [Internet]. Metabolism 2013;62:352–60. 22999011 10.1016/j.metabol.2012.08.005

[22] Sayiner M Lam B Golabi P. Advances and challenges in the management of advanced fibrosis in nonalcoholic steatohepatitis [Internet]. Therap Adv Gastroenterol 2018;11:1756284818811508. 10.1177/1756284818811508 PMC6243399 30479664

[23] Golabi P Sayiner M Fazel Y. Current complications and challenges in nonalcoholic steatohepatitis screening and diagnosis [Internet]. Expert Rev Gastroenterol Hepatol 2016;10:63–71. 26469309 10.1586/17474124.2016.1099433

[24] Younossi ZM Corey KE Alkhouri N. Clinical assessment for high-risk patients with non-alcoholic fatty liver disease in primary care and diabetology practices [Internet]. Aliment Pharmacol Ther 2020;52:513–26. 32598051 10.1111/apt.15830

[25] Lin ZH Xin YN Dong QJ. Performance of the aspartate aminotransferase-to-platelet ratio index for the staging of hepatitis C-related fibrosis: An updated meta-analysis [Internet]. Hepatology 2011;53:726–36. 21319189 10.1002/hep.24105

[26] Angulo P Hui JM Marchesini G. The NAFLD fibrosis score: A noninvasive system that identifies liver fibrosis in patients with NAFLD [internet]. Hepatology 2007;45:846–54. 17393509 10.1002/hep.21496

[27] Sterling RK Lissen E Clumeck N. Development of a simple noninvasive index to predict significant fibrosis in patients with HIV/HCV coinfection [Internet]. Hepatology 2006;43:1317–25. 16729309 10.1002/hep.21178

[28] Cholankeril G Wong RJ Hu M. Liver transplantation for nonalcoholic steatohepatitis in the US: Temporal trends and outcomes [internet]. Dig Dis Sci 2017;62:2915–22. 28744836 10.1007/s10620-017-4684-x

[29] Golabi P Bush H Stepanova M. Liver transplantation (LT) for cryptogenic cirrhosis (CC) and nonalcoholic steatohepatitis (NASH) cirrhosis: Data from the scientific registry of transplant recipients (SRTR): 1994 to 2016 [internet]. Medicine 2018;97:e11518. 30075518 10.1097/MD.0000000000011518 PMC6081090

[30] Anstee QM Targher G Day CP. Progression of NAFLD to diabetes mellitus, cardiovascular disease or cirrhosis [Internet]. Nat Rev Gastroenterol Hepatol 2013;10:330–44. 23507799 10.1038/nrgastro.2013.41

[31] Wong RJ Tran T Kaufman H. Increasing metabolic co-morbidities are associated with higher risk of advanced fibrosis in nonalcoholic steatohepatitis [Internet]. PLoS One 2019;14:e0220612. 31369606 10.1371/journal.pone.0220612 PMC6675045

[32] Verma S Jensen D Hart J. Predictive value of ALT levels for non-alcoholic steatohepatitis (NASH) and advanced fibrosis in non-alcoholic fatty liver disease (NAFLD) [Internet]. Liver Int 2013;33:1398–405. 23763360 10.1111/liv.12226

