# Supplementary material for: Identification of High-Risk Patients With Nonalcoholic Fatty Liver Disease Using Noninvasive Tests From Primary Care and Endocrinology Real-World Practices
Source: Clin Transl Gastroenterol. 2021 Apr 6;12(4):e00340. doi: 10.14309/ctg.0000000000000340 (PMC8032357; doi:10.14309/ctg.0000000000000340)
Supplement: SUPPLEMENTARY MATERIAL [file ct9-12-e00340-s001.pdf]

Supplementary Table 1: Clinicodemographic characteristics of patients based on Propensity Score

Case-control by propensity score	Linked	Not linked	Prob	All
N	103	103		206
Male sex	53 (51.5%)	54 (52.4%)	0.89	107 (51.9%)
Age	68.3 ± 9.6	67.3 ± 10.0	0.66	67.8 ± 9.8
Race				
White	60 (58.3%)	61 (59.2%)	0.89	121 (58.7%)
Black	30 (29.1%)	29 (28.2%)	0.88	59 (28.6%)
Hispanic	4 (3.9%)	4 (3.9%)	1.00	8 (3.9%)
Asian	5 (4.9%)	5 (4.9%)	1.00	10 (4.9%)
Body Mass Index, kg/m²	31.4 ± 6.6	33.0 ± 7.0	0.12	32.2 ± 6.8
Linked	101 (98.1%)	0 (0.0%)	<.0001	101 (49.0%)
Diabetes	56 (54.4%)	59 (57.3%)	0.67	115 (55.8%)
Hyperlipidemia	91 (88.3%)	96 (93.2%)	0.23	187 (90.8%)
Hypertension	85 (82.5%)	91 (88.3%)	0.24	176 (85.4%)
History of Myocardial Infarction	9 (8.9%)	8 (7.8%)	0.77	17 (8.3%)
History of Stroke	4 (4.0%)	8 (7.9%)	0.23	12 (5.9%)
History of Congestive Heart Failure	1 (1.0%)	2 (1.9%)	0.56	3 (1.5%)
History of Cancer	24 (23.5%)	21 (20.6%)	0.61	45 (22.1%)
Albumin, g/dl	4.06 ± 0.34	4.02 ± 0.56	0.32	4.04 ± 0.46
Alanine aminotransferase, IU/L	32.1 ± 30.6	24.0 ± 16.2	0.41	28.0 ± 24.7
Aspartate aminotransferase, IU/L	32.1 ± 20.3	21.2 ± 8.2	<.0001	26.7 ± 16.4
Platelets, 10^3/UL	199.0 ± 44.7	260.7 ± 69.6	<.0001	229.8 ± 66.1
AST-to-platelet ratio (APRI)	**0.419 ± 0.282**	**0.214 ± 0.093**	**<.0001**	**0.317 ± 0.233**
Fibrosis-4 (FIB-4)	**2.07 ± 0.80**	**1.23 ± 0.50**	**<.0001**	**1.65 ± 0.79**
NAFLD Fibrosis Score	**0.382 ± 0.996**	**-0.514 ± 1.389**	**<.0001**	**-0.066 ± 1.287**

Abbreviations: APRI: AST-to-platelet ratio, BMI: body mass index; FIB-4: Fibrosis-4
